# P38 Lupus enteritis as a first presentation of systemic lupus erythematosus in a previously well patient: a very rare occurrence

**DOI:** 10.1093/rap/rkae117.069

**Published:** 2024-11-05

**Authors:** Stephanie Ling, Dipesh Vasant, Vidula Godhamgaonkar, Vishnu Jeyalan, Surabhi Wig

**Affiliations:** The University of Manchester, Manchester, United Kingdom; Manchester University NHS Foundation Trust, Manchester, United Kingdom; The University of Manchester, Manchester, United Kingdom; Manchester University NHS Foundation Trust, Manchester, United Kingdom; Manchester University NHS Foundation Trust, Manchester, United Kingdom; Manchester University NHS Foundation Trust, Manchester, United Kingdom; The University of Manchester, Manchester, United Kingdom; Bolton NHS Foundation Trust, Bolton, United Kingdom

## Abstract

**Introduction:**

Lupus enteritis is an anecdotally rare condition (with no formal estimates of incidence) which can pose a diagnostic challenge, especially when presenting without other lupus-related symptoms and signs. Here, we present an unusual case where lupus enteritis was the first manifestation of systemic lupus erythematosus (SLE).

**Case description:**

A 65-year-old South Asian female presented with a 6-month history of intermittent nausea, vomiting and abdominal pain, with the only systemic feature being worsening fatigue. She had no connective tissue disease features, previous miscarriage or venous thromboembolism (VTE). Over six months, she had multiple hospitalisations in different hospitals with recurrent attacks of episodic abdominal pain and vomiting, associated with migratory and reversible intestinal inflammation on serial imaging. Initial investigations revealed lymphopenia (0.99x10^9^/L) and hypoalbuminaemia (25 g/L).

During a two-month admission at our centre, she underwent detailed gastrointestinal investigations, including four abdomino-pelvic CT scans during transient episodes of pain and vomiting, showing diffuse wall thickening affecting different parts of the gastrointestinal tract at different times. Following discussion at specialist radiology and inflammatory bowel disease multidisciplinary meetings, these CT scans and an MRI small bowel study were confirmed to demonstrate a transient, migratory, reversible pattern of bowel inflammation affecting the ileum, jejunum and colon at different time points; there were no clinical, endoscopic, histological or imaging features of inflammatory bowel disease. Gastro-duodenal biopsies were normal and negative for amyloidosis and tuberculosis. Colonic biopsies excluded inflammation. C1 esterase inhibitory deficiency was excluded.

A vasculitis screen was negative, but immunology was positive for anti-nuclear and anti-chromatin antibodies, with positive crithidia, but normal dsDNA. C3 was normal, but C4 was low at 0.04 g/L. Urinalysis showed significant albuminuria (413.8 mg/mmol). Following multidisciplinary discussion between gastroenterology, clinical immunology and rheumatology, a diagnosis of SLE was made, with an initial presentation of lupus enteritis. She received a tapering dose of corticosteroids and hydroxychloroquine.

The patient suffered from a right-sided multi-segmental pulmonary embolus with infarction, but this was felt to be due to her nephrotic syndrome (negative anti-cardiolipin and β2-glycoprotein antibodies). Renal biopsy revealed class I lupus nephritis with podocytopathy. The patient was duly commenced on tacrolimus as a steroid-sparing agent.

**Discussion:**

Lupus enteritis is a rare, poorly-defined condition, with only small amounts evidence regarding treatment and management in the literature, although a narrative review of 58 papers has recently been published. However, the average time between diagnosis of SLE and presentation with lupus enteritis is around 44 months, meaning that isolated enteritis as a first presentation of SLE is extremely rare. Only two out of 150 patients reviewed by Janssens *et al* (from a single tertiary lupus centre) presented with enteritis prior to a diagnosis of SLE. Lupus enteritis is potentially serious if left untreated, and can lead to life-threatening complications such as intestinal necrosis and/or perforation.

This rare presentation highlights the importance of considering lupus enteritis in the differential in any patient presenting with non-specific gastrointestinal symptoms and a migratory pattern of intestinal inflammation on radiological investigations. This can enable prompt diagnosis and timely management.

**Key learning points:**

• Lupus enteritis can be a first presentation of SLE without any other classical manifestations.

• Lupus enteritis is extremely rare, but can be life-threatening if not identified and rapidly treated.

• Radiology can demonstrate transient, migratory bowel inflammation on serial imaging.

